# The endogenous retrovirus ENS-1 provides active binding sites for transcription factors in embryonic stem cells that specify extra embryonic tissue

**DOI:** 10.1186/1742-4690-9-21

**Published:** 2012-03-15

**Authors:** Anne Mey, Hervé Acloque, Emmanuelle Lerat, Sébastien Gounel, Violaine Tribollet, Sophie Blanc, Damien Curton, Anne-Marie Birot, M Angela Nieto, Jacques Samarut

**Affiliations:** 1 Institut de Génomique Fonctionnelle de Lyon, Université de Lyon, Université Lyon 1, CNRS, INRA, Ecole Normale Supérieure de Lyon, 46 allée d'Italie, 69364 Lyon Cedex 07, France; 2 Instituto de Neurociencias de Alicante, CSIC-UMH, Avda. Ramón y Cajal s/n, 03550 Sant Joan d'Alacant, Spain; 3 Laboratoire de Genetique Cellulaire-INRA, ENVT, Chemin de Borde Rouge BP52627, 31326 Castanet Tolosan, France; 4 Université de Lyon, Lyon, F-69003, France; université Lyon 1, Villeurbanne, F-69622 cedex, France; CNRS, UMR5558, Laboratoire de Biométrie et Biologie Evolutive, 43 bd du 11 Novembre 1918, Villeurbanne F-69622 cedex, France

## Abstract

**Background:**

Long terminal repeats (LTR) from endogenous retroviruses (ERV) are source of binding sites for transcription factors which affect the host regulatory networks in different cell types, including pluripotent cells. The embryonic epiblast is made of pluripotent cells that are subjected to opposite transcriptional regulatory networks to give rise to distinct embryonic and extraembryonic lineages. To assess the transcriptional contribution of ERV to early developmental processes, we have characterized *in vitro *and *in vivo *the regulation of ENS-1, a host adopted and developmentally regulated ERV that is expressed in chick embryonic stem cells.

**Results:**

We show that *Ens-1 *LTR activity is controlled by two transcriptional pathways that drive pluripotent cells to alternative developmental fates. Indeed, both Nanog that maintains pluripotency and Gata4 that induces differentiation toward extraembryonic endoderm independently activate the LTR. Ets coactivators are required to support Gata factors' activity thus preventing inappropriate activation before epigenetic silencing occurs during differentiation. Consistent with their expression patterns during chick embryonic development, Gata4, Nanog and Ets1 are recruited on the LTR in embryonic stem cells; in the epiblast the complementary expression of Nanog and Gata/Ets correlates with the *Ens-1 *gene expression pattern; and Ens-1 transcripts are also detected in the hypoblast, an extraembryonic tissue expressing Gata4 and Ets2, but not Nanog. Accordingly, over expression of Gata4 in embryos induces an ectopic expression of *Ens-1*.

**Conclusion:**

Our results show that *Ens-1 *LTR have co-opted conditions required for the emergence of extraembryonic tissues from pluripotent epiblasts cells. By providing pluripotent cells with intact binding sites for Gata, Nanog, or both, *Ens-1 *LTR may promote distinct transcriptional networks in embryonic stem cells subpopulations and prime the separation between embryonic and extraembryonic fates.

## Background

Long terminal repeats (LTR) from endogenous retroviruses (ERV) are remnants of transposable elements disseminated in the genome that contain promoter activity [1] and can control nearby genes in different organisms [2-5]. They represent a source of binding sites for transcription factors [6], and some that are active in embryonic stem (ES) cells have been shown to rewire the Nanog and Oct3/4 transcriptional networks in a species-specific manner [7]. Whether these changes are neutral or reflect species-specific adaptation to conserved developmental processes is not known, but ERV that escape silencing in pluripotent cells have been described in several species [4,8].

ES cells are isolated from the inner cell mass of very early embryos and can generate all the cells of an organism [9], a unique property called pluripotency that is supported by Oct3/4 [10], Sox2 [11] and Nanog [12] transcription factors. Oct3/4 and Nanog inhibit differentiation toward embryonic and extraembryonic lineages, the latter providing nutrient exchange and inductive signals for the embryo [13]. These functions are well conserved in ES cells from different species, including chicken [14]. *In vivo*, the emergence of extraembryonic tissues from pluripotent cells represents the first cell fate decision and precedes the differentiation of the embryonic lineages. Notably in different species, Nanog deficiency makes the cells tolerant to differentiation into extraembryonic endoderm lineages [15-17] allowing the action of Gata-6 [18] and Gata-4 [19,20] transcription factors to drive extraembryonic endoderm formation. However, it is not clear what mechanisms guide pluripotent cells toward embryonic or extraembryonic lineages upon the suppression of the controls exerted by Oct3/4 [21] and Nanog [15].

To better understand the contribution of LTR to the transcriptional networks available in ES cells, we focused our interest on a developmentally regulated ERV and characterized its transcriptional regulation. The *Ens-1 *LTR controls the expression of a multigenic family of genes of retroviral origin, *ENS *(Embryonic Normal Stem cell), present only in Galliform species. The *Ens-1 *copy presents the most complete coding region and has been maintained in Galliform genomes through negative selection pressure [22] as observed for host-adopted retrotransposons [23]. *Ens-1 *also called *Erni*, is expressed in pluripotent cells of the epiblast and later in the prospective neural plate [24,25], where it has been demonstrated to delay the expression of Sox2 [26] affecting the timing of emergence of the definitive neural plate and thus embryonic patterning. *In vitro, Ens-1 *is expressed in chicken ES (cES) cells [25] and is repressed when ES cells differentiation is induced, mimicking the repression of the *Ens-1 *LTR as further development occurs [27]. In addition to the coding regions, more than 800 copies of solo-LTR are disseminated and placed in close contact to host genes in sense or in anti-sense orientations [22] where they might act as alternative promoters [28]. We show here that the *Ens-1 *LTR is under the control of both Nanog and Gata factors in such a way that may direct the formation of the extraembryonic endoderm when ES cells exit pluripotency.

## Results

### A cooperation between distinct DNA motifs controls the activity of the *Ens-1 *promoter

**Figure 1:**
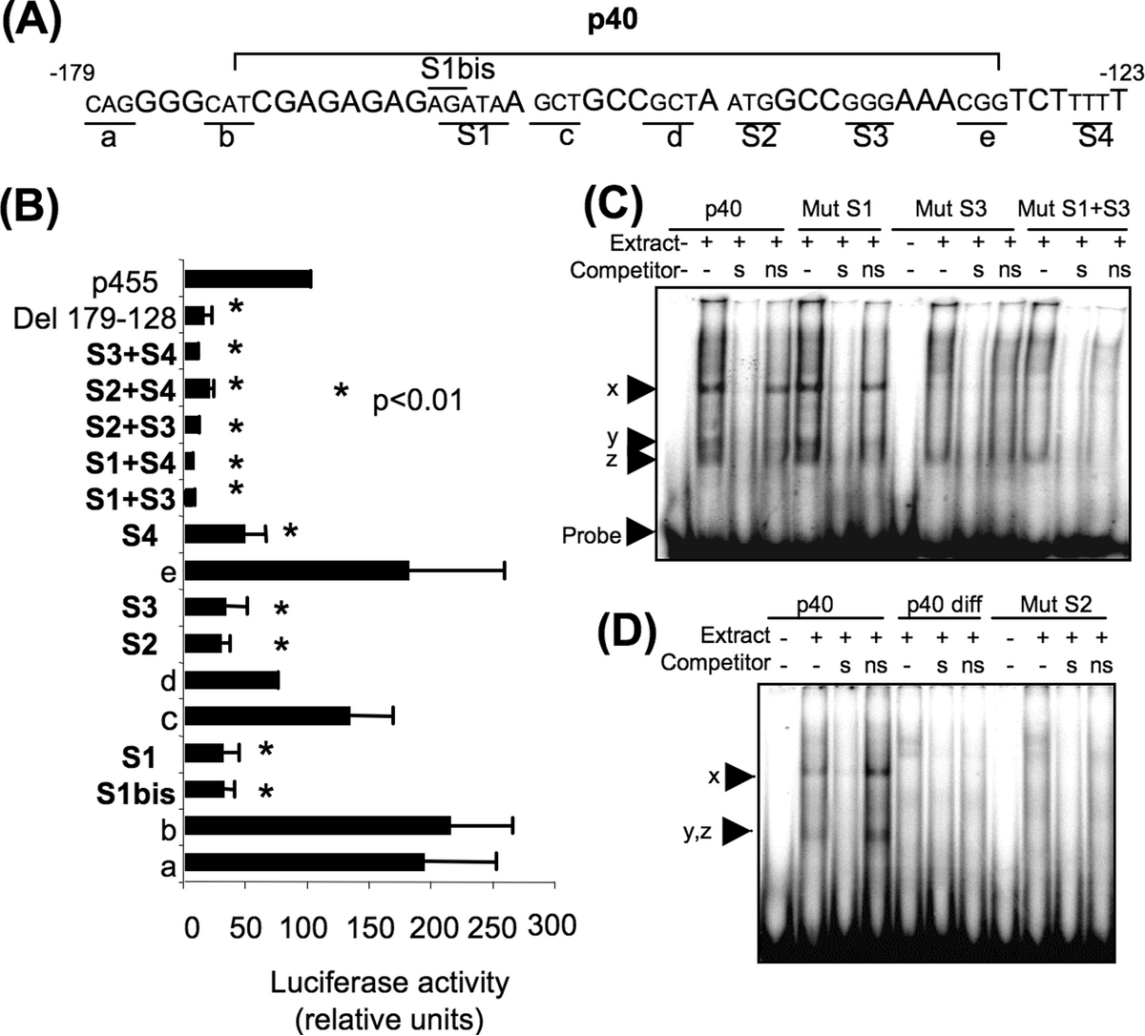
**Four domains cooperate to fully control the p455 promoter activity in cES cells**. (**A**) Sequence of the active domain in the p455 luciferase-reporter construct. Deletions performed between positions -179 and -123 of the p455 promoter relatively to the transcription start site are indicated in reduced characters, underlined and called a, b, c, d, e, S1, S2, S3, S4. The sequence of the p40 oligonucleotide used as a probe in EMSA experiments, is bounded above. (**B**) Luciferase activity from p455 constructs carrying the mutations presented in (**A**). Mutations inhibiting the activity are designed S1 to S4 while the others are called a to e. All luciferase activities were normalized by co-transfection of cES cells with a CMV-renilla luciferase reporter and results are the means of three independent experiments +/- s.d. Statistics are the results of a t test relatively to the values obtained with the non mutated p455 construct. **(C) **EMSA were performed with p40 or with the mutated p40 labelled probes (MutS1: GAGCG in place of AGATA; MutS3: AAA in place of GGG) using nuclear extracts from cES cells. **(D) **EMSA with p40 probe using nuclear extracts from cES cells or from cES cells induced to differentiate for 4 days with retinoic acid (p40 diff). Mut S2 probe (GCA in place of ATG) was used with cES extracts. The position of the DNA-protein complexes **x, y **and **z **or the probe used alone are indicated with arrows. For each probe competition, experiments were performed with a 100-fold molar excess of the same unlabelled nucleotide for specific specific binding (s lanes) or an unrelated nucleotide for nonspecific binding (ns lanes). Results are representative of two others.

The promoter activity of *Ens-1 *in the chicken ES (cES) cells is supported by a 455 bp sequence (p455) upstream of the transcription initiation site and isolated from the U3 domain of the whole LTR [25]. The transcription factor CP2 that partially controls p455 activity binds to a domain located within the already explored 277 bp of the 5' end of this region [27]. Deletions within the first 237 bp of p455 placed upstream of a firefly luciferase reporter gene have allowed us to find major determinants for the promoter activity between the positions -179 and -128 (Additional file 1: Figure S1). To reveal active binding sites for transcription factors, a series of site-directed deletions were performed in this region (Figure 1A). Four sites, S1 to S4, contributed to *Ens-1 *promoter activity, and single deletions of each of them were not sufficient to abrogate the promoter activity to the same extent as the whole -179/-128 deletion (90% inhibition) (Figure 1B). No inhibition was obtained after site directed deletions downstream of position -128, including those in two putative [27] Gata binding sites (Additional file 1: Figure S1). These results suggest that several elements cooperate for the full promoter activity, as confirmed by the analysis of combined mutations of these four sites which inhibited the activity of the p455 construct with the same efficiency as a large deletion.

Site S2 was separated from site S3 by only three nucleotides suggesting a close contact between factors occupying both sites and a narrow sequence specificity of the inhibiting deletions when compared with other close mutations.

It thus seems that the *Ens-1 *promoter is controlled by a combination of DNA binding proteins recognizing distinct and specific motifs and acting in a synergistic manner to promote the transcriptional activity.

### The active domains are differently involved in the recruitment of ES specific protein complexes on the promoter

To address the role of the DNA active motifs in the recruitment of protein complexes, electrophoretic mobility shift assays (EMSA) were performed with nuclear extracts from cES cells. A ^32^P end-labelled double stranded oligonucleotide, spanning sites S1 to S3 and called p40, was used as a probe and was compared with the same sequence carrying mutations on sites S1, S2 or S3 (Figure 1A). To compare probes of similar sizes, the mutations were made by substitution. The p40 probe formed three different protein-DNA complexes, **x, y **and **z **(Figure 1C), the latest being sometimes resumed to a single band as illustrated in Figure 1D. These complexes were competed off by a 100-fold molar excess of unlabelled probe, but only the complex **x **resisted to a 100-fold molar excess of an irrelevant unlabelled probe indicating specific binding only for **x **(Figure 1C). When using nuclear extracts from cES cells induced to differentiate for four days with retinoic acid, no complex was formed on the DNA probe (Figure 1D) indicating that the binding was specific for pluripotent stem cells. As shown in Figure 1C the mutation of the S1 site did not affect complex formation, while mutation of the S3 site inhibited the formation of the complex **x**. The double mutation in S1 and S3 gave similar results as those obtained with the single S3 mutant version, confirming that S1 was not involved in complex formation. The mutation S2 (Figure 1D) completely inhibited the formation of the complex.

Altogether, these results indicate that a protein complex is specific for undifferentiated cES cells bound to the functional domain of the *Ens-1 *promoter and that sites S2 and S3 were necessary for the interaction with DNA.

**Figure 2:**
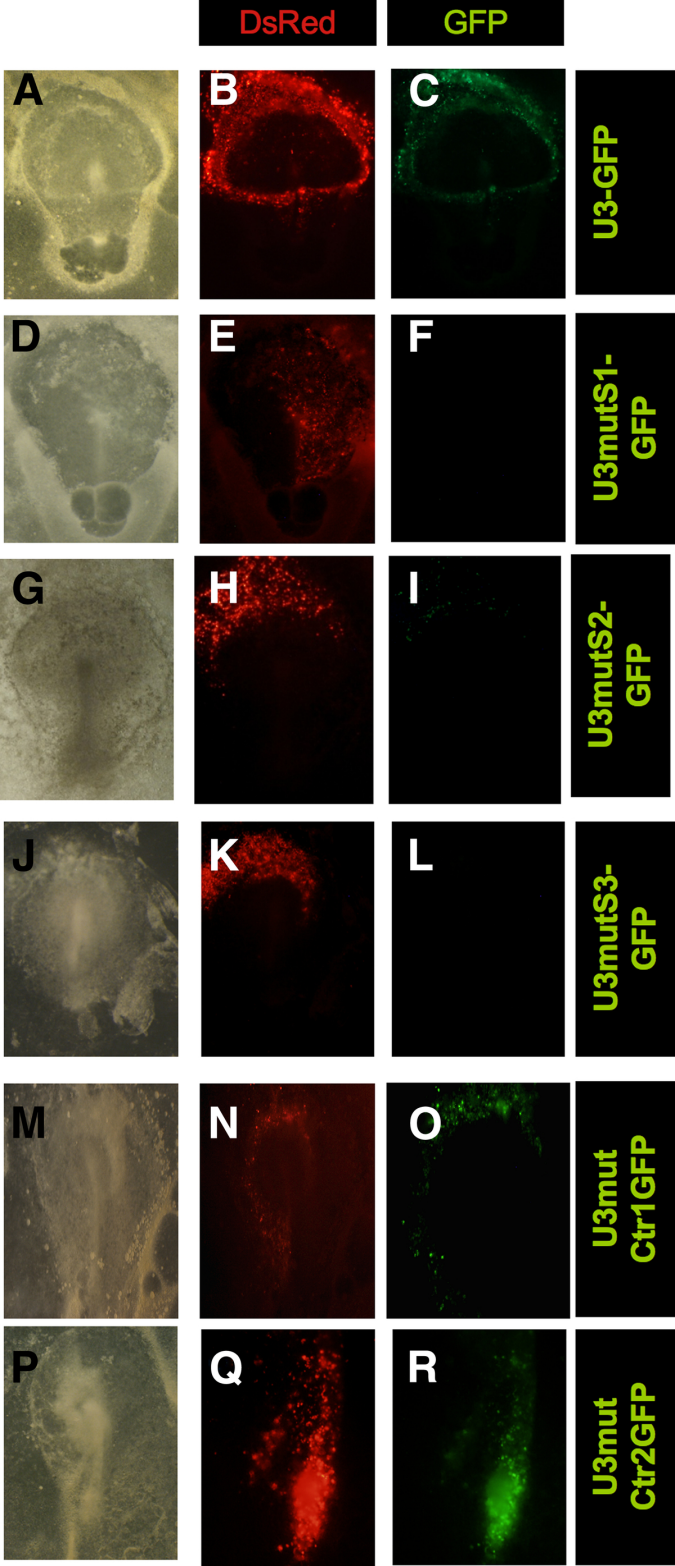
**The activity of *Ens-1 *promoter *in vivo *involves similar domains as in cES cells**. Epiblast cells of pre-primitive streak chicken embryos were cotransfected by electroporation with CMV-DsRed (used to visualize the transfected cells) and U3-GFP vector, in which U3 contains the whole promoter of *Ens-1 ***(A-C)**. GFP expression was compared with U3 constructs presenting the MutS1 **(D-F)**, the MutS2 **(G-I)**, or the MutS3 **(J-L) **mutations (see Figure 1 legend). As negative controls, mutations on non-functional sites were performed upstream of site S1 (Mut Ctr1: deletion of site b, Figure 1A) **(M-O) **or at position -129 (Mut Ctr2: deletion of the GTGTG motif) **(P-R)**. The same results were obtained in three independent experiments.

*In vivo*, mutations in S1 and S3 of the full length promoter contained in the U3 domain from the *Ens-1 *LTR [25] totally abrogated promoter activity (Figure 2), confirming the importance of these DNA sequences in the regulation of the whole promoter. Activity was also strongly inhibited by mutation in site S2 but a very low and residual activity was still detected confirming the involvement of additional motifs (Figure 2). Therefore, the activity of the *Ens-1 *promoter was based on similar motifs in embryonic stem cells and in the early embryonic tissues and albeit with different contributions, all S1 to S3 motifs are necessary to sustain the activity of the whole promoter.

### Identification of the transcription factors recruited on the active binding sites

**Figure 3:**
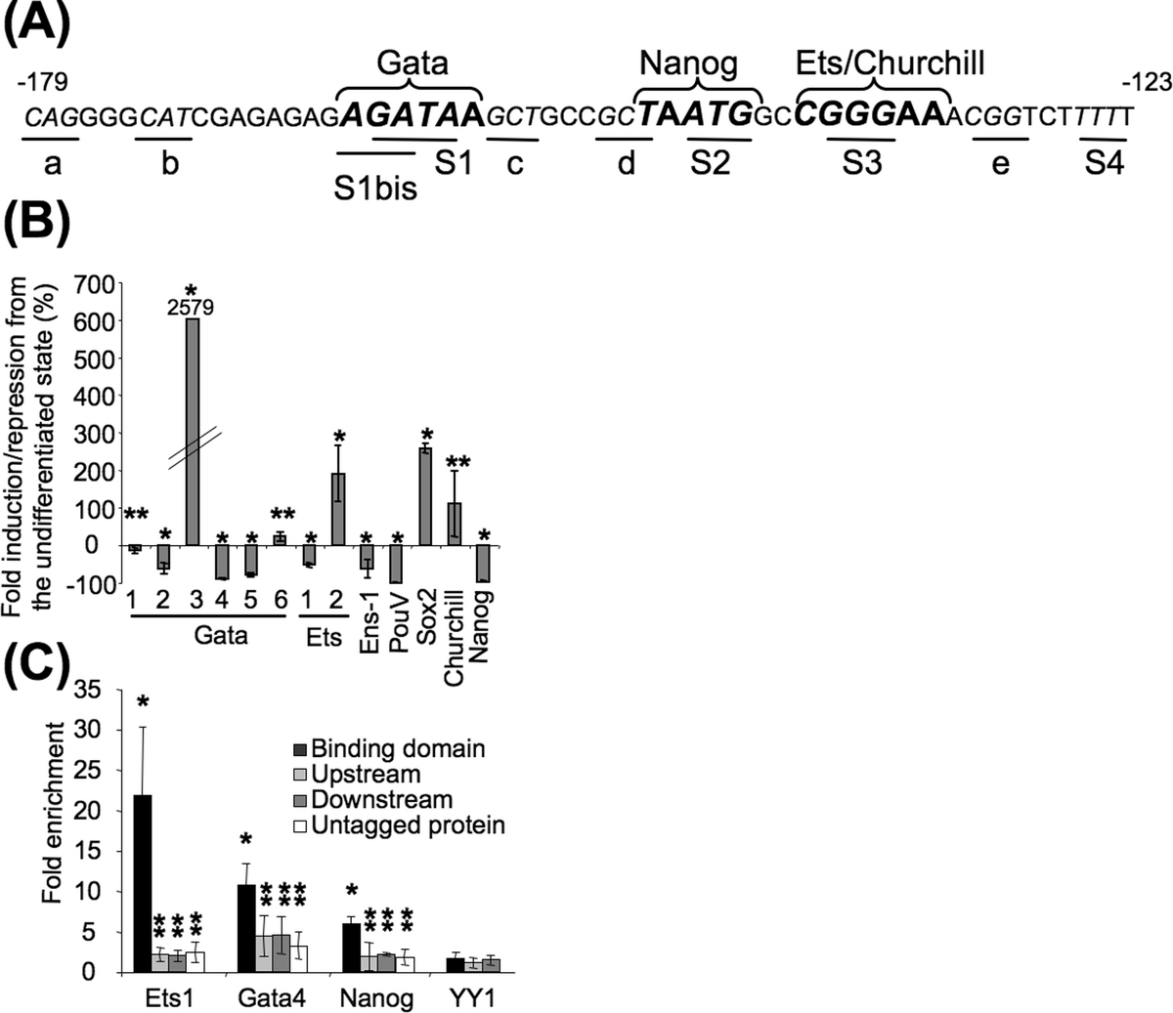
**Nanog, Ets1 and Gata4 interact with the functional domain of the *Ens*LTR promoter in cES cells**. **(A) **Binding sites for Gata, Nanog, Ets and Churchill transcription factors in the functional domain of the *Ens-1 *LTR are represented in bold and upper characters. The mutations used in Figure 1 are indicated by underline and italic characters. **(B) **Real time PCR analysis performed on cDNA from cES induced to differentiate 48 h with retinoic acid. Expression levels of the indicated transcripts are represented as percentages of the levels obtained in undifferentiated cES. The numbers that are underlined identify the member of the Gata or of the Ets transcription factors family indicated below. Means are from three experiments +/- s.d. For Gata3 s.d. is +/- 1097. T test is relative to the values obtained in undifferentiated cells: *p < 0.05, **p > 0.05. (C) Chromatin-immunoprecipitation of the *Ens-1 *LTR promoter with anti-flag antibodies in cES transfected with expression vectors encoding for Nanog, Gata4 or Ets1 proteins in fusion with a flag tag. cES transfected with untagged transcription factors were used as negative controls. Results obtained with the irrelevant transcription factor YY1 in fusion with a flag tag are also represented. Results represent the fold enrichment compared to cells transfected with empty vector. The non-specific binding of the tagged transcription factors on upstream and on downstream regions is represented as well as the non specific interaction of the untagged transcription factors with the binding domain. Means of at least two experiments +/- s.d. are shown. T test is relative to the values obtained with the tagged YY1 transcription factor, *p < 0.05, **p > 0.05.

**Figure 4:**
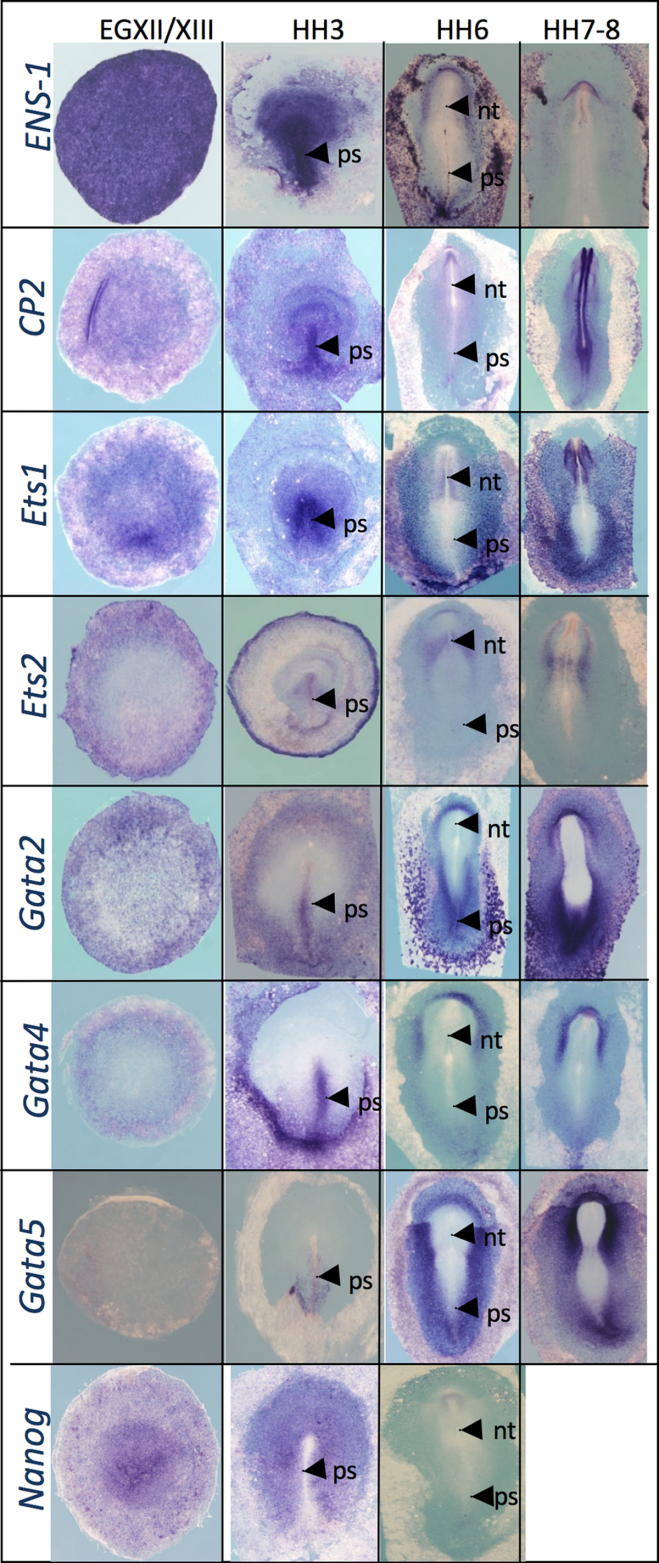
**Expression patterns of *Ens1, CP2, Ets1, Ets2, Gata2, Gata4, Gata5 *and *Nanog *during early chicken embryo development detected by in situ hybridization**. Dorsal view of preprimitive streak embryos (Stage EG XII/XIII), primitive streak embryos (stage HH3) and early neurulas (stages HH6-8) are shown. Embryos are oriented with rostral side toward the top of the page and the dorsal side up. ps: primitive streak, nt: notochord.

The Match, Patch and AliBaba2 programs were used to help in the identification of transcription factors that may recognize the promoter active sites (see Figure 3A). The S1 site was recognized as a potential binding site for the family of Gata transcription factors (A/T)GATA(A/G) [29] while the site S3 overlapped the core motif GGAA recognized by the Ets 1/2 transcription factors [30] and by the Churchill transcription factor (CGGGAA) expressed in the developing nervous system of chick [31] (Figure 3A). The complete motif for Nanog binding (TAATGG) [32] was also present in the *Ens-1 *promoter and encompasses the S2 motif (Figure 3A). No homology with known transcription factor binding sites was found for S4. Alternatively, this motif may indirectly increase transcription by favouring the accessibility of flanking DNA to transcription factors as described for polydT tracts [33]. To identify which of these factors may promote the specific activity of the promoter in ES cells and in the early embryo, their expression patterns were checked by QPCR analysis (Figure 3B) and in situ hybridization (Figure 4). As expected *PouV*, the chicken homolog of *Oct4 *[14], and *Nanog *were fully repressed in cES cells induced to differentiate by retinoic acid, while *Sox2 *that is not expressed before the epiblast is fated to become neural plate in the chicken [34] was strongly induced, even more than *Churchill *(Figure 3B). With respect to the transcription of the six Gata family members, no variation was observed for *Gata1 *and *Gata6 *mRNA; *Gata3 *mRNA was induced while *Gata2, Gata4 *and *Gata5 *were repressed during differentiation. Similarly, *Ets2 *was reproducibly induced during the differentiation while *Ets1 *was repressed (Figure 3B). Overall these results show that all the candidates for binding to the S1, S2 or S3 sites could be detected in cES cells, but the only ones that were reproducibly repressed during differentiation were *Nanog, Ets-1 *and members of the Gata family, *Gata2, Gata4 *and *Gata5*. *In situ *hybridization experiments in whole embryos (Figure 4) showed that all these factors, except Gata5, were detected in the epiblast at stage XII/XIII from which ES cells were derived. Ets2 was also detected at this stage. At gastrulation, *Gata *and *Nanog *showed complementary expression patterns within the ectoderm (HH3, Figure 4), and their combined expression coincided with that of *Ens-1*. Similarly, *Ets1 *and *Ets2 *also showed complementary transcription patterns until stage HH3 (Figure 4).

To confirm the recruitment of these transcription factors to the promoter, we performed chromatin immunoprecipitation (ChIP) assays using cES cells expressing exogenous Ets1, Gata4 or Nanog in fusion with a flag tag. Untagged factors and a flag-tagged version of the ubiquitous YY1 transcription factor were used as negative controls. Results shown in Figure 3C confirmed the specific recruitment of all the tagged proteins on the regulatory domain of the *Ens-1 *promoter when compared with flanking regions. These interactions were significantly higher than those obtained with negative controls. Altogether these data indicate that Nanog, Gata4, and Ets1 expressed in ES cells and repressed upon differentiation are recruited to the *Ens-1 *promoter in pluripotent cells and are good candidates to support its activity.

### Combinations with Ets are required for Gata or Nanog to support the full *Ens-1 *promoter activity

**Figure 5:**
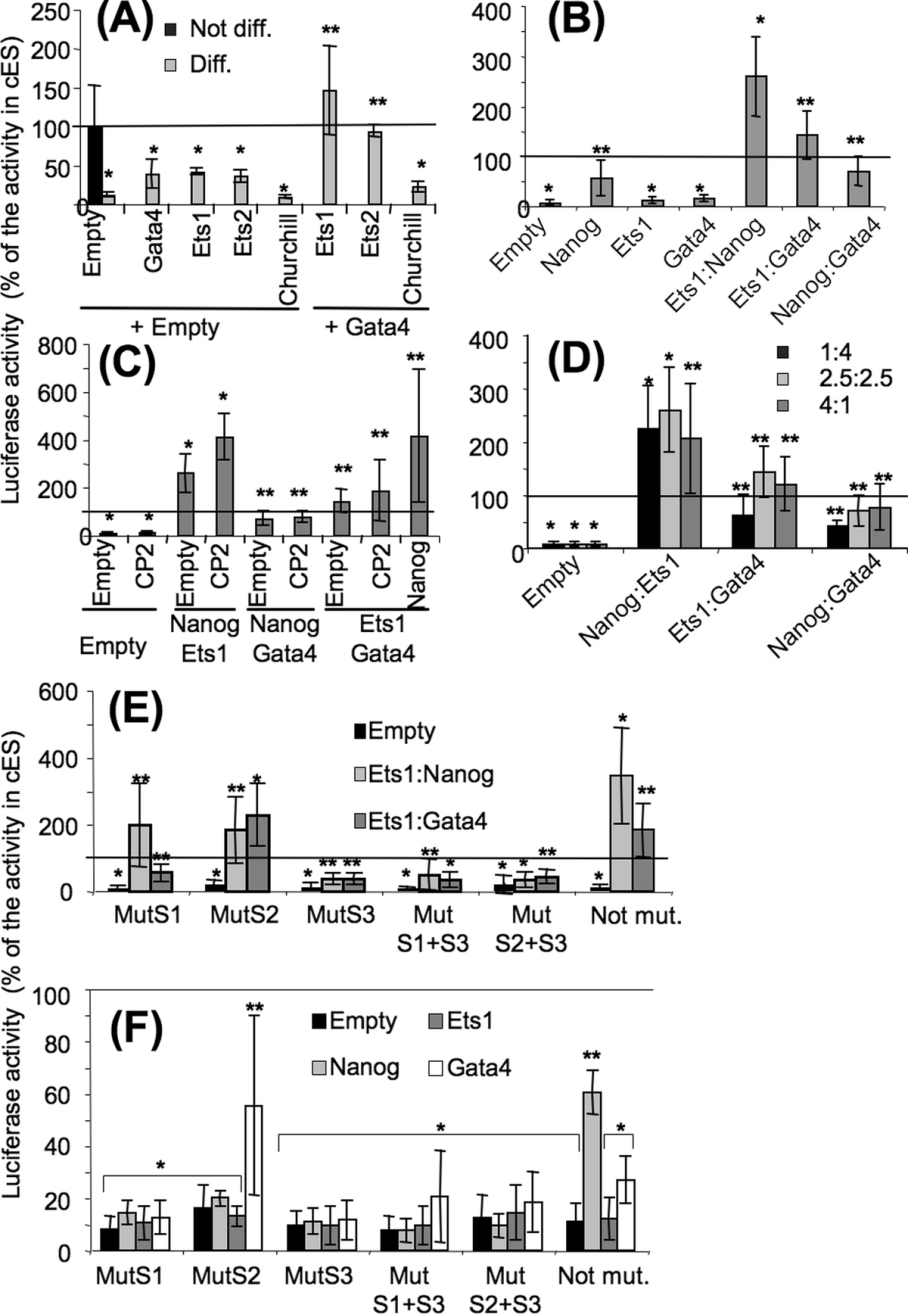
**Ets factors synergize with Nanog and are indispensable for Gata4 to restore the activity of the p455 promoter in differentiated cells**. **(A, B, C, E, F) **cES cells induced to differentiate 48 h with retinoic acid (Diff) were transfected with the p455-Luc vector and with pCi-neo vectors expressing the indicated transcription factors tested in combinations or alone. Equal quantities of each vector were transfected. **(D) **Co-transfections were performed as in **(A) **but with unequal quantities of each vector. The proportion of each is indicated as parts of five in the figure in the order indicated on the abscissa. **(E) **Combinations of transcription factors restoring the promoter activity in differentiated cells were tested on p455-Luc mutated on one or two of the activation sites. **(F) **Effect of mutations on the activity mediated by each transcription factors transfected alone in differentated cells. All luciferase activities were measured 24 h after transfection and are represented as percentages of the value obtained with p455-Luc in cES cells co-transfected with empty vectors (Not Diff.). Results are the means of at least three independent experiments +/- s.d. In all the figures, t test values are relative to the results obtained in undifferentiated cES cells with p455-Luc: *p < 0.05, **p > 0.05. For (**E**) only, t test values relatively to the p455-Luc activity in differentiated cells transfected with a given combination of transcription factors have also been calculated and are indicated in the text.

The respective roles of Ets and Gata factors in the control of the *Ens-1 *promoter were evaluated in differentiated cells which are devoid of Nanog (see Figure 3B) and in which the promoter activity is repressed [25,27]. Indeed, ES cells induced to differentiate with retinoic acid lost about 90% of *Ens-1 *promoter activity, which was not restored by the ectopic expression of any of the factors alone (Figure 5A). However combinations of Ets-1 or -2 with Gata4 induced promoter activity. The transcription factor Churchill, used instead of Ets due to its potential interaction with the same DNA motif, failed to synergize with the Gata factors (Figure 5A). Thus the combination of Ets and Gata4 is necessary and sufficient to support the *Ens-1 *promoter activity in differentiated cells. Some redundancy was observed between the members of the same family independently of their regulation during differentiation (Additional file 2: Figure S2). The endogenous expression of the Ets and Gata family members in differentiated cells may explain the activation of the *Ens-1 *promoter by Gata4, Ets1 and Ets2 transfected separately. However, the best results were obtained with Gata/Ets combinations, particularly involving either Ets1 or Gata4 (Figure 3B) which are limiting in differentiated cells (see Figure 3B). Both Ets1 and Gata4 were also tested for their complementarity with Nanog (Figure 5B). Nanog alone strongly induced promoter activity in differentiated cells when compared with Gata4 or Ets1. Nanog combined with Ets1 reached levels of promoter activity that exceeded even those obtained in undifferentiated cES cells. In contrast no synergy was observed with Gata4 suggesting that Ets1 was independently required for both Gata4 and Nanog activities (Figure 5B). When Ets1 was mixed with both Gata4 and Nanog (Figure 5C), the activity of the promoter was induced at higher levels than those observed with Nanog/Ets1 but with a strong variability between experiments as also observed with Gata4/Ets1 (Figure 5A). Interestingly, in the absence of Ets1, the addition of Gata4 to Nanog was not a source of variability in the transcriptional activation of p455. In contrast, the addition of the CP2 transcription factor enhanced the response to Nanog/Ets1 without increasing the variability, but was neutral when the promoter was placed under the control of Gata4/Ets1 (Figure 5C). These data confirmed that CP2 is an enhancer of *Ens-1 *promoter activity in ES cells [27]. To better characterize the additive effect of transcription factors, we compared the influence of equal quantities or a 4-fold excess of one of the two co-transfected factors. In both Gata4/Ets1 and Nanog/Ets1 combinations, the results were mainly influenced by a decrease in the Ets1 level (Figure 5D) but with different outcomes. Limiting Ets1 impaired the activity of the Gata4/Ets1 dimer. This was not the case with Nanog as this combination still restored promoter activity but with a higher variability between experiments. Altogether these results indicate that both Gata4 and Nanog are involved in promoter activity in ES cells and that both cooperate with Ets1 in an independent manner.

To test whether the active motifs in the *Ens-1 *promoter that have been characterized in ES cells were also used in differentiated cells, the combinations that fully restored the promoter activity were tested on p455 and on its mutated versions. As expected, the activation mediated by Gata4/Ets1 or by Nanog/Ets1 was fully impaired by mutations affecting either S1 + S3 or S2 + S3, respectively, leading to significantly lower luciferase activities than those obtained in undifferentiated cells with the wild type promoter. None of the single mutations abrogated the activity obtained with combinations of the transcription factors to the same extent. Indeed, with any of the transcription factors combinations tested, luciferase activities of p455 with single mutations were either not significantly different or higher than those observed in undifferentiated cells with p455 (Figure 5E). However, for a given combination of transcription factors, strong differences in the contribution of each binding site were revealed when compared to the activity on p455 in differentiated cells (Figure 5E).

As expected, the mutation in S3 (in an Ets binding site) significantly inhibited the activity mediated by Gata4/Ets1 or by Nanog/Ets1 on the wild type promoter (*p *< 0.05) while the S1 mutation (in a Gata binding site) only inhibited (*p *< 0.05) the activity of the Gata4/Ets1 combination (Figure 5E). In contrast, the S2 mutated version (in a Nanog binding site) did not show significant inhibition of the activity (*p *> 0.05) after ectopic expression of Nanog/Ets1 (Figure 5E) suggesting an indirect effect of Nanog in this combination of exogenous factors. To clarify this result, we evaluated the consequences of the mutations on promoter activity when expressing only Nanog.

Results shown in Figure 5F indicate that any of the single or double mutations significantly impaired the reactivation of the promoter mediated by Nanog, confirming the indirect effect and the requirement for intact S1, S2 and S3 binding sites. Results were different when expressing the other transcription factors. Although Gata4 alone failed to reactivate the p455 construct (Figure 5A and 5F), it reactivated the S2 mutant version to levels comparable to those obtained with the intact promoter in undifferentiated cells (Figure 5F). This was not the case with Nanog alone that restored the promoter activity on wild type p455 only. This indicates that the indirect effect of Nanog on the promoter activity required its interaction with the S2 site, probably releasing another DNA binding protein acting as a repressor. EMSA experiments in human HEK293 cells confirmed the direct interaction of the transfected chicken protein Gata4 with S1 site in non-ES cells (Additional file 3: Figure S3).

In contrast to Gata4, Ets1 was not able to increase the activity of a S2 mutant version of the promoter, indicating that the activity mediated by the combination of Nanog and Ets1 could not be only due to an indirect effect. The 2.5 (Figure 5B) to 4 (Figure 5E) fold increase in promoter activity in differentiated versus undifferentiated cells further illustrates the synergy between Nanog and Ets1.

Altogether these results reveal a functional interplay between Gata4, Nanog and Ets1 that requires intact S1, S2 and S3 binding sites, respectively. They also show the major role played by Ets1 to promote the activity mediated by Gata4 and by Nanog and also that Nanog partly acts in an indirect manner, probably by competition with another DNA binding protein acting as activity repressor on the S2 site.

### Gata4 induces the ectopic expression of *Ens-1 *in the developing embryo and is associated with the expression of *Ens-1 *in extraembryonic tissues

**Figure 6:**
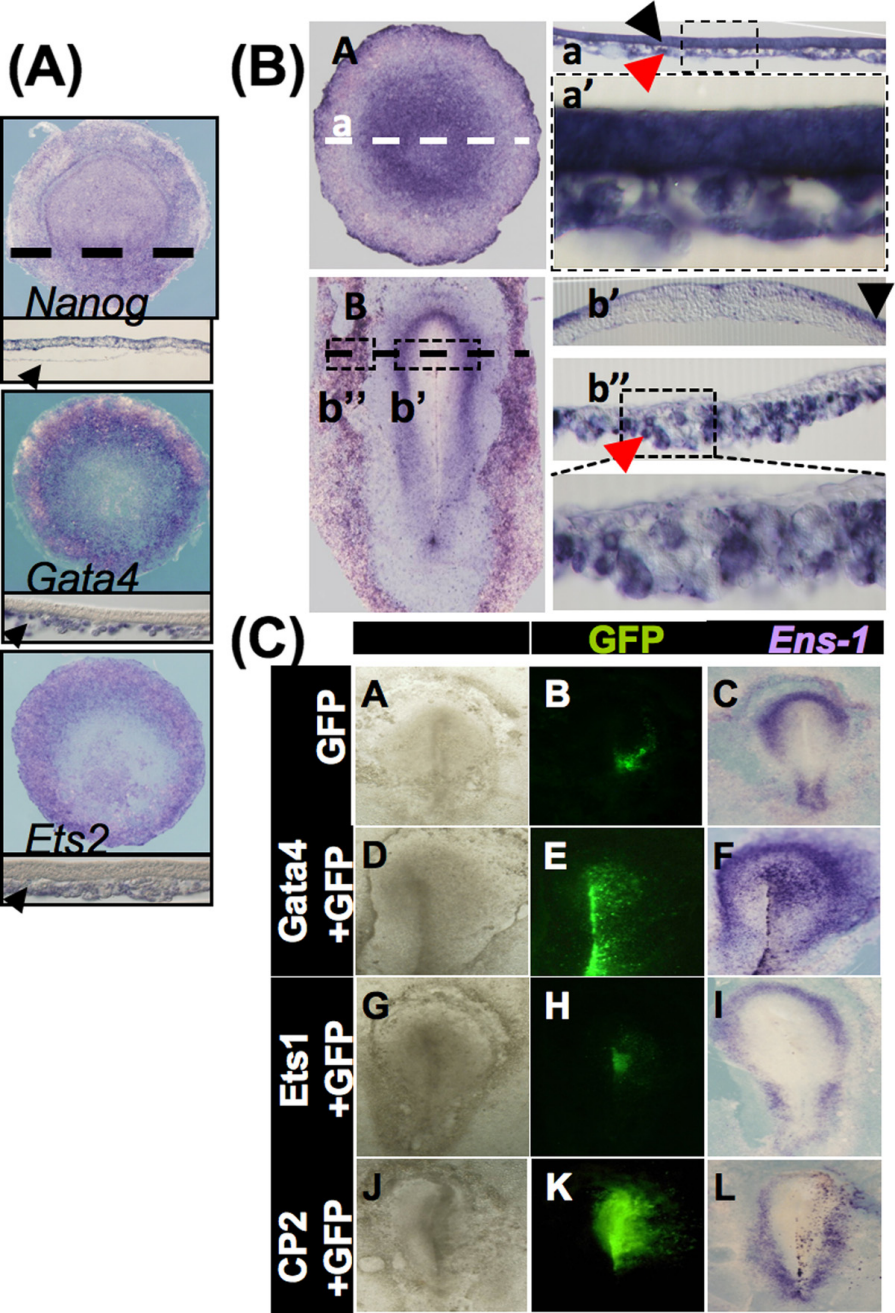
**Gata4 induces the expression of *Ens-1 in vivo***. **(A) ***Nanog, Gata4 *and *Ets2 *transcripts detected by in situ hybridization in stage XIII (EG) chick blastula. Sections highlight specific expression either in the epiblast or in the hypoblast (black arrow). **(B) ***In situ *hybridization in chick stage XIII (EG) blastula A and in stage 5 (H&H) gastrula B. Sections show *Ens-1 *expression both in the epiblast (black arrow on a, enlargement on a') and in the hypoblast (red arrow in a, enlargement on a') at blastula stages, and later, at gastrulation, at the border of neural and non neural ectoderm (black arrow on b') and in the extraembryonic mesoderm (red arrow on b", enlargement below). Dotted lines indicate sections' level. **(C) **Epiblasts of preprimitive streak chicken embryos were electroporated for the ectopic expression of GFP alone **(A-C) **or conjointly with the transcription factors Gata4 **(D-F)**, Ets1 **(G-I) **and CP2 **(J-L)**. *Ens-1 *transcripts were detected ten hours later by *in situ *hybridization. Results obtained in 10 out of 19 electroporated embryos with Gata4 **(F)**, in 4 out of 17 with CP2 **(L)**, in 13 out of 13 with Ets1 **(I) **and in 12 out of 12 **(C) **with GFP alone.

Nanog and Gata6/Gata4 exert opposite influences on mammalian pluripotent cells toward the epiblast and the extraembryonic endoderm respectively [35]. In the chick embryo the hypoblast is equivalent to the mouse anterior visceral endoderm that derives from the extraembryonic endoderm [36]. The hypoblast expressed Gata4 and Ets2, but not Nanog (Figure. 6A). In agreement with our results in differentiated cells, *in situ *hybridizations in whole embryos revealed the expression of *Ens-1 *in the hypoblast in addition to the embryonic epiblast (Figure 6B). Altogether these results are compatible with a role for Gata4 in the maintenance of the *Ens-1 *LTR activity in extraembryonic tissues.

To confirm that Gata and Ets factors can support the activity of the *Ens-1 *promoter *in vivo*, Gata4 and Ets1 were overexpessed by electroporation in stage HH3 chick embryos. Interestingly, Gata4 electroporation, but not Ets1, was sufficient to induce *Ens-1 *ectopic expression (Figure 6C). Since Gata4 could not induce *Ens-1 *promoter activity when used alone in differentiated cells, these results may be due to the endogenous expression of Ets1 and Ets2 at the electroporation site (see Figure 4) that can cooperate with Gata4. In contrast, Gata factors were not expressed at the electroporation site but rather overlapped with *Ens-1 *expression (see Figure 4), discarding the possibility of testing the activity of Ets1 ectopically. These results confirmed that Gata4 is an activator of the *Ens-1 *LTR *in vivo *acting independently from Nanog. The electroporation of CP2 poorly induced the ectopic expression of *Ens-1 *confirming that its enhancer activity mainly depends of the control exerted by Nanog that was repressed at that stage (see Figure 4).

**Figure 7:**
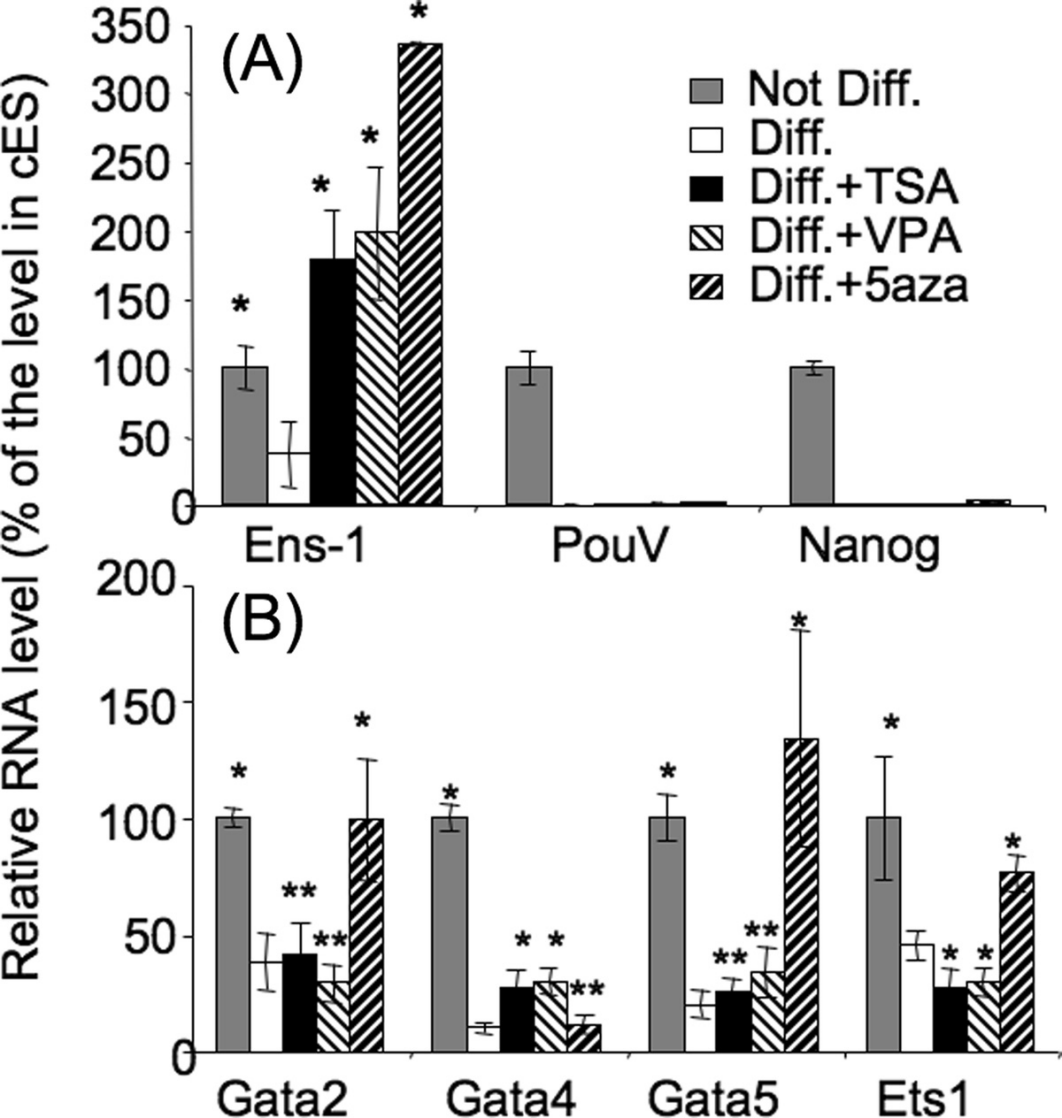
**Epigenetic regulations silence the *Ens-1 *LTR in differentiated cells**. cES induced to differentiate 48 h with retinoic acid (Diff) were further treated with TSA (Trichostatin A, 10 nM), VPA (Valproic acid, 100 μM) or 5aza (5-aza-cytidine, 100 μM) in differentiation medium without retinoic acid for 12 h. **(A) **Expression levels of Ens-1, PouV and Nanog transcripts measured by real time PCR. **(B) **Expression levels of the Gata and Ets1 transcripts that are repressed during differentiation. Results from three experiments +/- s.d. are represented as percentages of the value obtained in undifferentiated cells (Not Diff). T test: *p < 0.05, **p > 0.05 relative to the values obtained in untreated differentiated cells.

Surprisingly, overexpression of Gata/Ets failed to reactivate the expression of *Ens-1 *in ES cells induced to differentiate in vitro with retinoic acid. To assess whether epigenetic regulations were responsible for this lack of response, differentiated cells were treated with drugs that change the chromatin status such as histone deacetylase inhibitors (valproic acid and trichostatin A) or a DNA methyl transferase inhibitor (5-aza-cytidine). These treatments were sufficient to fully reactivate and even reach higher levels of *Ens-1 *transcripts when compared to the expression in ES cells (Figure 7A). These drugs did not restore the expression of *PouV *and *Nanog *excluding that our results were an indirect consequence of cell reprogramming. Histone deacetylase inhibitors did not restore the expression of *Gata4, Ets1*, or any of the Gata factors repressed during differentiation (Figure 7B) indicating that the transcription factors present in differentiated cells were sufficient to activate the promoter.

These results are in agreement with the existence of an additional level of regulation that involves epigenetic silencing, thereby restricting the accessibility of *Ens-1 *LTR for the binding of transcription factors. This is likely to occur at developmental stages where Ens-1 is no longer necessary and is in fact not expressed.

### Distribution of the active copies of the *Ens-1 *promoter in the chicken genome

**Figure 8:**
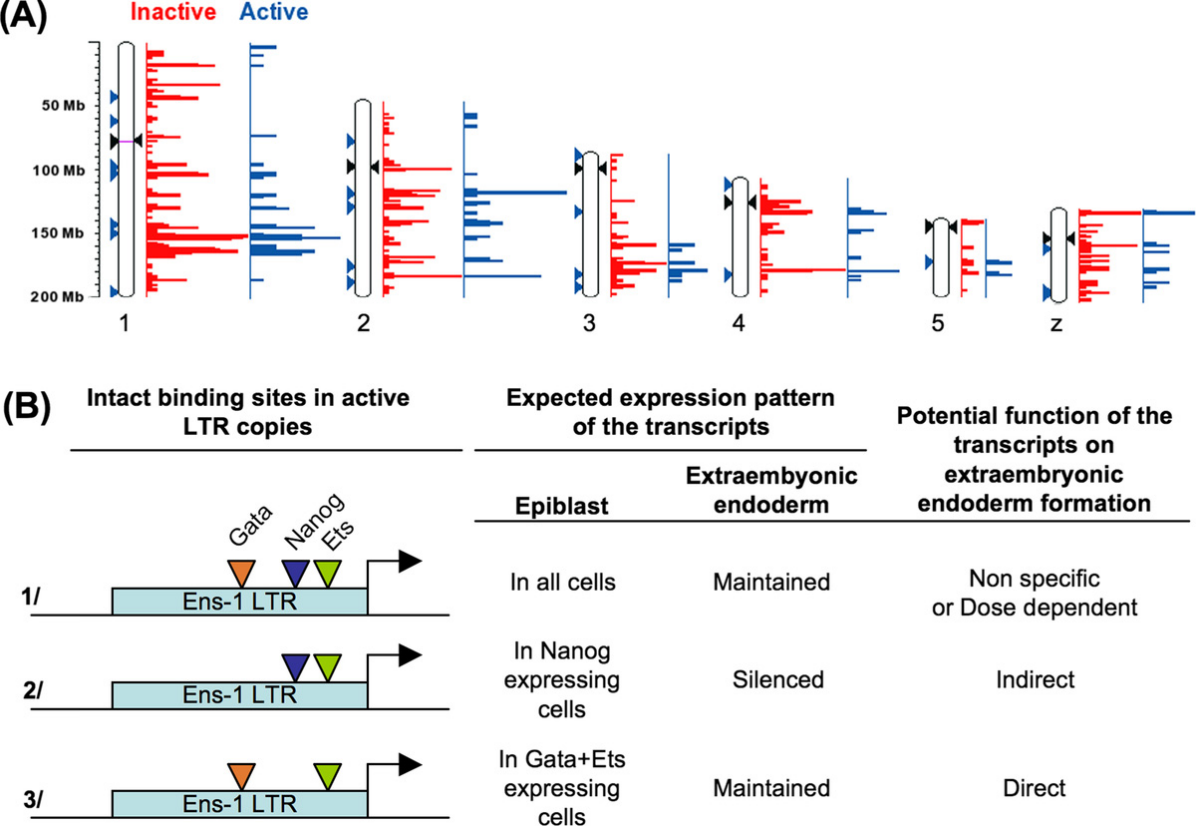
**Representation of the active copies of the *Ens-1 *LTR on the main chicken chromosomes and potential consequences**. **(A) **Density along the chromosomes 1 to 5 and z, of active solo-LTR (in blue) and inactive solo-LTR (in red) based on the sequence of the four activation motifs. The blue arrows indicate individual active solo-LTR inserted near genes. Distance in Megabases is indicated on the left scale. The black arrows indicate the position of each centromere. The purple line on the chromosome 1 indicates the position of the Nanog gene. **(B) **Combinations of intact binding sites that recruit transcription factors and may support LTR activity. The expected expression pattern in the epiblast and in the hypoblast is indicated for the transcripts induced by each type of LTR. Functional interpretation of each expression pattern is also mentioned. In situation 2, LTR activity is increased by Ets binding but depends on Nanog.

The characterization of *Ens-1 *LTR allowed us to perform a new analysis of their distribution on the latest version of the chicken genome considering only copies that contain the intact S1, S2, S3 and S4 sites required for full promoter activity. A total of 227 potentially active solo-LTR were revealed. Among them, 44 were located at less than 20 kb from genes or inside genes (list of 71 genes available upon request), and the rest were classified as intergenic. It is worth noting here that an intact TATA box-like sequence (GATAAAA) [27] was found in 200 out of the 227 (88%) potentially active solo-LTR and in 40 out of the 44 mentioned (91%) that are located close to genes. Therefore most of the LTR with four intact activation domains may support direct transcription. Most of them (225 out of 227, 99%) also have an intact CP2 binding domain (CNRG-N6-CNRG) shown in a previous paper to support the enhancer activity of the *Ens-1 *promoter [27]. This observation also supports a role for the active LTR in the transcriptional regulation of more distant sequences. The different insertions located on the main chromosomes (1,2,3,4,5 and z) were represented for active and inactive copies (solo-LTR with at least one mutation in the motifs S1 to S4) (Figure 8A). Both were located in the same clusters except for those in the regions surrounding the centromeres where we found the inactive but not the active solo-LTR. This was particularly true for chromosomes 1, 2, 3 and 5. These results suggest a non-random distribution of the active and inactive solo-LTR regarding heterochromatin enriched regions. Interestingly, on chromosome 1, the pluripotency gene *Nanog *was located near the centromere and at distance from active solo LTR. No insertion was found on chromosome 17, which contains the *PouV *gene that sustains cES pluripotency [14]. This suggests that pluripotency supporting genes are preserved from the influence of *Ens-1 *LTR, in agreement with a putative role for the *Ens-1 *LTR in the control of host genes exerted either in *cis *or in *trans*. To address whether the nearby genes belong to a particular functional category, gene ontology annotations of the genes were retrieved from the Ensembl database using the Biomart tool [37]. The most represented categories were genes whose products are associated with the membrane (about 25%) and the intermediate filaments components (approx. 12%) as previously observed with other adopted repetitive elements [38]. LTR with four intact motifs may support transcription in all the cells of the epiblast and later maintain expression in the hypoblast (Figure 8B situation 1/) as observed for *Ens-1 *although at different levels (see Figure 6B panel A), perhaps reflecting differences in transcription factor concentrations. This heterogeneity in the epiblast may account for the emergence of cells forming the extraembryonic endoderm or may reflect the association with a maintained or a delayed function. Interestingly, some nearby genes of solo-LTR have already been involved in embryonic or extraembryonic development such as Klf-6 [39], teneurin-4 fragment [40] or the conserved microRNA miR-7b involved in the inhibition of Fos [41] that is required for extraembryonic endoderm epithelial organization [42]. Alternatively, LTR copies presenting only Nanog (Figure 8B situation 2/) or Gata/Ets (Figure 8B situation 3/) binding motifs would show a more restricted transcriptional pattern in the epiblast. These situations are compatible with the priming of neighbouring genes for future silencing or with their subsequent role as cells exit pluripotency as summarised in Figure 8B presenting three potential functional situations. Importantly, they are all consistent with a role of *Ens-1 *LTR in the first developmental decisions through the production of differentially regulated transcripts in pluripotent cells.

## Discussion

This study addresses the question of whether the activity of an ERV LTR at very early developmental stages might be a source of targeted variability in the regulatory pathways operating in pluripotent cells. We show that the balance between the two opposite regulatory pathways controlling the decision between embryonic and extraembryonic tissues is co-opted in the regulation of the *Ens-1 *LTR promoter and correlates with its pattern of transcriptional activity.

We show that in ES cells, the *Ens-1 *LTR is controlled by the pluripotency specific transcription factor Nanog as described for other ERV that escape silencing [7]. However, this LTR is also activated by a combination of Gata and Ets transcription factors, two families that are not restricted to pluripotent cells, but whose members were found here to be expressed in ES cells and in the epiblast in agreement with a previous report [43]. At early developmental stages, the expression pattern of Gata and Ets transcription factors is likely to promote that of the ERV gene *Ens-1*, even in the absence of Nanog, such as in the primitive streak. In agreement with this observation, electroporation of Gata4 induced ectopic expression of *Ens-1 *in embryonic tissues that expressed Ets1 but not Nanog. In mammals, Gata4 overexpression is sufficient to transform ES cells into the extraembryonic endoderm lineage [20], and in agreement with this, we found expression of Gata4 in the chick hypoblast along with that of Ets-2. We show here that *Ens-1 *was also expressed in this extraembryonic tissue. In post-streak embryonic tissues, a good correlation was also observed between *Gata4 *and *Ens-1 *expression patterns until the formation of the neural plate (HH6), but Ets factors were not detected anymore suggesting the involvement of additional regulators. Indeed, in differentiated cells the activation domain attributed to Nanog was shown to play an indirect role in the activity mediated by Gata4 in the absence of Ets1. At later developmental stages *Ens-1 *is repressed in the whole embryo [24,25] despite the wider expression of Gata [44,45] and Ets [46] factors. In accordance with the silencing mechanisms reported for other ERV [4,8], epigenetic regulations are likely to restrict the promoter accessibility in irrelevant tissues as observed here in differentiated cells where the expression of *Ens-1 *was strongly induced by treatment with epigenetic modifying drugs but not by the overexpression of Gata/Ets. Our results thus reveal that the Gata/Ets combination plays a role at developmental stages that correlates with the emergence of the hypoblast while distinct mechanisms are likely to occur in the neural plate [24,47] and later.

Several lines of evidence indicate that both Nanog and Ets/Gata regulations are active in ES cells. First, all were expressed in ES cells; secondly, we found that Nanog, Gata4 and Ets1 were recruited on the *Ens-1 *promoter active domain in ES cells; thirdly, the suppression of the Nanog binding site only partially suppressed the promoter activity in ES cells and reciprocally with mutations in the Gata or in the Ets binding sites; fourthly, the activity mediated by Nanog is increased by Ets-1. This dual regulation is in agreement with the recent demonstration in the mouse that Nanog is required for the extraembryonic endoderm formation [48] that relies on the heterogeneity of ES cells populations. Accordingly distinct levels of the pluripotency marker Nanog are found among individual ES cells, Nanog-low cells expressing higher levels of extraembryonic endoderm markers [49] and being more prone to differentiate [50]. Similarly the epiblast that concentrates pluripotent cells, also contains cellular sub-populations expressing extraembryonic endoderm markers in different species [51,52]. These cells are likely precursors of the extraembryonic endoderm. Heterogeneity in the expression levels of Nanog, Ets and Gata factors was also observed here in stage XII/XIII chick epiblast where the periphery of the embryo that concentrates Gata factors expressed lower levels of Nanog transcripts. Despite this heterogeneity in the distribution of its regulating transcription factors, the expression level of *Ens-1 *was maintained in the whole epiblast probably reflecting the ability of both regulation pathways to support the *Ens-1 *LTR activity in cells with distinct fates. In embryonic tissues, Ens-1 regulates the timing of *Sox2 *activation and thus the emergence of the definitive neural plate [26]. The present data support an earlier role of *Ens-1 *or co-regulated sequences in the process that drives the formation of the hypoblast from pluripotent cells. Accordingly, *Ens-1 *expression was found to be maintained in the hypoblast.

Experiments performed in differentiated cells revealed that Nanog as well as the Gata4/Ets1 combination can restore the promoter activity normally observed in ES cells. However, additional regulations are likely to favor one or the other regulation pathway as illustrated by the transcription factor CP2 that is an enhancer of the *Ens-1 *promoter activity in ES cells [27], but was shown here to solely promote the activation mediated by Nanog.

This balance between opposite active binding sites provided by the *Ens-1 *LTR is thus coherent with the requirements supporting the emergence of the extraembryonic endoderm from pluripotent tissues and may contribute to this progress. Active copies of the *Ens-1 *LTR may support in pluripotent cells the specific priming of genes involved later in the extraembryonic endoderm or in the neural plate formation. This is illustrated with *Ens-1 *that is expressed in cES cells but involved later during the neural plate formation [26]. Alternatively the epigenetic silencing of the *Ens-1 *LTR during differentiation may serve to repress irrelevant genes [53,54] according to the heterochromatin formation during ES cells differentiation [55].

Genome-wide studies combined with transcriptome analysis have concluded that LTR promoters may have an impact on tissue specific transcription [56,57]. We show here that only 26% (227 on 874 [22]) of the *Ens-1 *LTR copies in the chicken genome contain an intact activation domain that supports transcriptional activity in the early embryo. Most of them are far from genes, and one third is localized in or at less than 20 kb of host genes, a distance that is compatible with a promoter activity and may direct gene functions in specific tissues. They may also act as enhancers inducing the transcription in both orientations [25] of non-coding RNA from intergenic loci [58]. Such sequences are known to be important players during development [59] and may be involved in the guidance of chromatin-modifying complexes on specific targets [60] as required during ES cells differentiation [61-63]. The presence of active LTR near genes already involved in embryonic or in extraembryonic development as listed here is in favour of relevant species-specific adaptations.

## Conclusion

Our results demonstrate that the *Ens-1 *LTR support gene expression in pluripotent and in extraembryonic tissues thus providing conditions for cell priming compatible with the unclarified emergence of extraembryonic endoderm cells from ES cells. In addition to *Ens-1*, transcriptome analysis based on active LTR distribution along the genome will serve as a basis to explore their contribution in the regulation of other genes and their role in defining ES cells subpopulations with distinct cell fates.

## Methods

### Cell culture and DNA transfection

The culturing of cES cells and their differentiation by retinoic acid 10^-6 ^M have been previously described [27]. cES cells or cES cells induced to differentiate 48 hours by retinoic acid were transfected using Lipofectamine 2000 reagent (Invitrogen) with p455-Firefly Luciferase reporter construct containing the *Ens-1 *minimal promoter, the Renilla Luciferase reporter construct, pRL-CMV, to normalize for transfection efficiency and expression vectors for transcription factors used alone or in combinations. Except when mentioned, an equal quantity of each expression vector was used and total DNA quantity between conditions was maintained constant using empty vector. Twenty-four hours after transfection the Firefly and Renilla Luciferase luminescences were successively measured using Dual Luciferase Assay (Promega) as described by the manufacturer. Firefly reporter gene values were normalized to the activity of the Renilla luciferase. Chromatin modifying drugs Trichostatin A (TSA), Valproic acid (VPA) and 5-azacytidine (5-aza) were from Sigma.

HEK (Human Embryonic Kidney) 293 cells were from ATCC (CRL-1573) and cultivated in DMEM (Gibco) supplemented with 10% fetal bovine serum (Perbio) as recommended by the supplier.

### DNA binding assays

The preparation of DNA binding assays and nuclear extracts was performed as previously described [27] using double-stranded DNA probes labelled with ^32^P^-^ATP (Amersham). For competition experiments, a 100-fold molar excess of unlabelled double-stranded nucleotide, synthesized by Sigma, was incubated for 10 min with nuclear extract prior to the addition of the labeled double-stranded probe. For supershift experiments in HEK293 cells anti-Flag M2 antibody was from Sigma. Whole IgG purified from mice were from Zymed.

### DNA constructs and site directed mutagenesis

The luciferase reporter construct p455-Luc was done using the pGL2 basic vector (Promega) as previously described and includes the LTR sequence from -455 to +83 of the transcription start site [27]. Targeted mutagenesis by deletions was performed using the Quick Change Mutagenesis Kit (Stratagene) following the manufacturer's instructions. The U3-GFP construct expresses a GFP reporter gene placed under the control of to the sequence from -738 to +83 of the transcription start site described before [25]. This largest construct contains the 738 bp of the promoter defined previously [27] which is present in the U3 region of the LTR of *Ens *genes [25]. Both p455-Luc and U3-GFP contain 83 bp from the R region 5' end starting downstream the transcription start site [25].

Site directed mutations were performed by replacements or by deletions of bases as indicated in the figure legends. Large deletions in the constructs p455 del 128-87, del 128-57 del 179-128, del 179-87, del 179-57 and del 237-31 were obtained by ligation of two PCR amplified fragments surrounding the deletion. The transcription factors were cloned in a pCi-Neo expression vector (Promega) modified to introduce a flag tag at the N-terminal part of the protein. Ets-2 (Genbank:X07202), Gata3 (Genbank:XM_417294), Gata4 (Genbank:XM_420041) and Gata5 (Genbank:NM_205421) were amplified from chicken ES cells cDNA. Chicken Ets-1 (p54, Genbank:X13026) was given by Dr B. Wasylyk [64], Churchill (Genbank:AF238863) was a gift from Dr C. Stern [31]. For over-expression experiments in the embryo, transcription factor coding sequences were placed under the control of the CAG promoter (a chicken b-actin promoter combined with CMV enhancer) to ensure strong expression [65]. The predictions for transcription factor binding sites were carried out using the indicated programs available online (http://www.gene-regulation.com/pub/programs.html).

### Real time PCR

RNA was extracted using an RNeasy kit with on-column DNase digestion (Qiagen). Reverse transcription was carried out with 1 μg of RNA and SuperScript III (Invitrogen). Real-Time PCR was performed using the MXP-300P PCR-system (Stratagene), Mix-Quantitect SYBR Green (Qiagen) as reagent under the following cycling conditions: 40 cycles at 95°C for 30 seconds, at 55°C for 1 minute and 72°C for 30 seconds. Samples were run in duplicate and gene expression levels were calculated using Delta Ct (http://www.gene-quantification.info/) normalized with the chicken 40S ribosomal protein S17 as housekeeping gene. The primers used are listed in Table 1.

**Table 1:** Oligonucleotides used for gene expression analysis

Gene	Sense (5'-3')	Anti-sense (5'-3')
RS 17*	ACACCCGTCTGGGCA ACGACT	CCCGCTGGATGCGCTTCATCA
Gata1	CACTGCACTCCGACATCCA	GTACCAAGATCCCACAGTCCTT
Gata5	CTTCCATTACGAACTCAGACAGCAC	GGACACCGACACAATGCCTTG
Gata6	GAATTCAGACGAGGAAACGAAAACC	ACGTAGATGTTGGAGTCATAGGAAC
Ets-1	CCAGCTTCATCACAGAGTCCTACC	AGGGATAGTCGTTCTCGTACTTGAG
Ets-2	CAGAGGAATGCTCAAGCGGC	GCACTTCCTGGAGCGTTTGA
Churchill	ATCATCACCTACGACCACCTG	CAGGGTTACAAACTGCCTTCA
Sox2*	GCAGAGAAAAGGGAAAAAGGA	TTTCCTAGGGAGGGGTATGAA
Nanog*	CAGCAGACCTCTCCTTGACC	TTCCTTGTCCCACTCTCACC
PouV*	GTTGTCCGGGTCTGGTTCT	GTGGAAAGGTGGCATGTAGAC
Ens-1	CACCAGTCAGGACCCAAAGT	GGGGATGAAACCTTTTTGGT

Primers for *gallus *Gata2, Gata3 and Gata4 were purchased from Qiagen

* Primers previously described [64]

### Chromatin immunoprecipitation

ES cells were transfected using Lipofectamine 2000 (Invitrogen) with pCi vectors encoding for the indicated transcription factor in fusion with a Flag tag. The following day, cells were fixed with formaldehyde, collected and lysed in 50 mM Tris-HCl (pH 8.1), 10 mM EDTA, 0.5 mM EGTA, 1% (w/v) SDS, and proteases inhibitor for 5 minutes on ice. The cells were then sonicated to shear chromatin to a final average size of 200-600 base pairs. Sonicated chromatin was diluted 1/10 with the following buffer: 20 mM Tris-HCl (pH 8.1), 1% (v/v) Triton X-100, 150 mM NaCl, 1 mM EDTA and proteases inhibitors. Immunoprecipitation was carried out overnight at 4°C with 50 ml of agarose beads coated with anti-Flag M2 antibody (Sigma). Beads were washed extensively, and bound material was eluted by two incubation rounds of 15 minutes in 1% (w/v) SDS and 0.1 M NaHCO3 at room temperature. Cross-linking was reversed by incubation 4 h at 65°C in 200 mM NaCl and 100 mg/ml proteinase K. The DNA was purified using Mini-Elute columns (Qiagen). Aliquots were used for quantitative real-time PCR as described above. The oligonuclotides used were designed either inside the p455 region (GAGGAACAAGTCCAGGCAAG; GATGGCCATTTTCCTTGAGA), or 1000 bp upstream (CCCACGGTACACAATGAACA; GCTAGGGAGCCCTTTAACCA), or 1000 bp downstream (TGGTGTGGTGTTTGCAGTTT; CCCTTTGTTGAGGAAAGCAC) from the *Ens-1 *copy on chromosome 5. The region bounded by the primers designed inside the p455 sequence is located between positions -278 and -93 from the transcription start site.

### Embryos

Fertilised chicken eggs were purchased from Granja Santa Isabel, Cordoba, Spain. Eggs were incubated, opened and staged according to Eyal-Giladi and Kochav [66] for the pre-primitive streak stages and Hamburger and Hamilton [67] for subsequent stages. They were dissected and fixed overnight in 4% paraformaldehyde in phosphate buffered saline (PBS) at 4°C.

### Chicken embryos electroporation

Stage 2-4 [67] chick embryos were explanted, washed in PBS, and placed upside down over an electroporation chamber (NEPAGEN) containing a platinum electrode connected to the negative pole. After visualizing the embryo, a solution containing expression plasmids (2 mg/ml in PBS with 0.1% Fastgreen and 6% sucrose) was injected between the vitelline membrane and the epiblast. An anodal electrode was placed over the hypoblast to cover the injected area and contact was made with PBS. A train of electric pulses (5 pulses, 4 Volts, 50 ms, 0.5 Hz) were applied using an Intracept TSS10 pulse stimulator (Intracell). The embryos were then placed in culture as described [68] and allowed to develop until they reach the required stage. Embryos were then photographed with a Leica MZFLIII dissecting microscope to record GFP or DsRed expression and fixed overnight in 4% paraformaldehyde (PFA) in PBS at 4°C for subsequent processing.

### Whole mount in situ hybridization

Whole-mount in situ hybridization was carried out in chick embryos at various stages of development as previously described [69]. Digoxigenin-labelled probes for ENS1 [25], CP2 [27], Ets1, Ets2, Gata2, Gata4, Gata5 and Nanog with digoxigenin-UTP (Roche) were synthesized. After hybridization, embryos were fixed in 4% paraformaldehyde in PBS, washed in PBS, and photographed in whole-mount under a Leica M10 dissecting scope. Subsequently, some embryos were embedded in gelatine and sectioned in a vibratome at 40 μm. These slices were photographed using an Olympus DP70 digital camera mounted on a Leica DMR microscope with Nomarski optics.

### Detection and localization of solo-LTR in the chicken genome

Using the sequence of the *Ens-1 *LTR as a reference, the occurrence of solo-LTR were searched in the latest version of the chicken genome (release galGal3) that was downloaded from the UCSC website (http://hgdownload.cse.ucsc.edu/downloads.html) using blastn [70]. We classified each solo-LTR as active or inactive according to the presence or absence of the motifs identified as being essential to its activity by using the program fuzznuc from the EMBOSS package [71]. We thus obtained 227 active solo-LTR and 916 inactive solo-LTR. Using the Ensembl facilities [72] (http://www.ensembl.org/index.html), we mapped the distribution of each type of solo-LTR on the chromosomes. By combining the positions of the active solo-LTR and those of the genes, we determined the active solo-LTR inserted close to genes (at less than 20 kb or inside the genes). Genes ontology annotations were retrieved from the Ensembl database using the Biomart tool [37] (http://www.ensembl.org/index.html).

## Competing interests

The authors declare that they have no competing interests.

## Authors' contributions

Conceived and designed the experiments: AM. Performed the experiments: SG, VT, SB, DC, AMB, HA, EL AM. Analyzed the data: AM, HA, EL. Wrote the manuscript: AM. Revised the manuscript: HA, EL, JS, MAN. Gave final approval of the version to be published: AM, JS, MAN.

## Supplementary Material

Additional file 1 **Figure S1. The -179 to -128 bp residue upstream of the transcription initiation site is required for the p455 promoter activity**. (**A**) Schematic representation of the wild-type p455 reporter construct used in transfection experiments and position of the different deletion edges of the constructs used in (**B**). (**B**) cES cells were transfected with wild type or with one of the deleted p455 (p455 Del) luciferase reporters illustrated in (**A**). All luciferase activities were normalized by co-transfection with a CMV-renilla luciferase reporter. (**C**) The contribution of two putative Gata binding sites to the promoter activity of p455 was examined. Site directed deletions were performed in one of the following positions: Gata n°2: TATC -111/-114 or Gata n°3: TATC +47/+50. The deletion in site S1 described in Figure 1 and showing inhibition is used as reference. Luciferase activities obtained with these constructs in cES cells were compared to that obtained with wild type p455 as indicated in (**B**). Means are +/- s.d. of at least three independent experiments. Statistics are from t tests relative to the value obtained with p455. Click here for file

Additional file 2 **Figure S2. Redundancy between Gata factors to restore the activity of the p455 promoter in differentiated cells**. Experiments were performed as indicated in Figure 4 with equal quantities of pCi-neo vectors expressing the indicated transcription factors transfected in cES cells induced to differentiate 48 h with retonoic acid (Diff). Results are percentages of the value obtained with p455-Luc in cES cells transfected with empty vector. All the results are the means of three independent experiments +/- s.d. T test: *p < 0.05, **p > 0.05, relative to the values obtained in undifferentiated cells (Not diff.). Click here for file

Additional file 3 **Figure S3. Direct interaction of Gata4 with the site S1 of the promoter sequence**. HEK293 cells were transfected with expression vectors encoding for the Gata4 protein in fusion with a flag tag or untagged as control. Nuclear extracts were used for EMSA assays with the labelled p40 probe or with p40 probes mutated in sites S1, S2 or S3. Supershifts were performed using an anti-Flag antibody or whole IgG as control; both used at 1 μg per lane. On the right is represented the result obtained with untransfected HEK293 cells. This lane is from the same gel but moved from the opposite side. Results are from one experiment representative of two. Click here for file
