# Supplementary figures and images for: The endogenous retrovirus ENS-1 provides active binding sites for transcription factors in embryonic stem cells that specify extra embryonic tissue
Source: Retrovirology. 2012 Mar 15;9:21. doi: 10.1186/1742-4690-9-21 (PMC3362752; doi:10.1186/1742-4690-9-21)

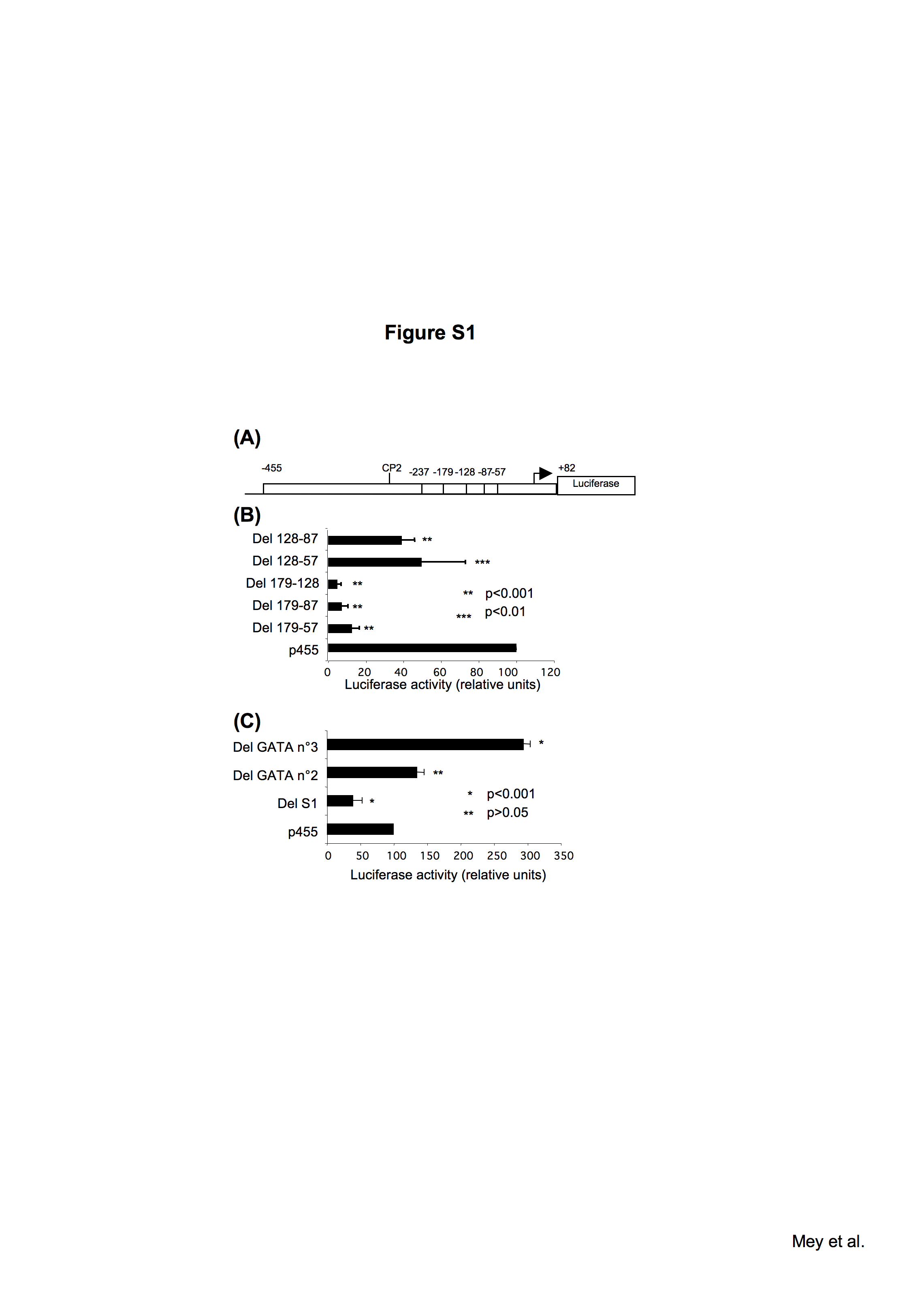

Supplement: Additional file 1 — Figure S1. The -179 to -128 bp residue upstream of the transcription initiation site is required for the p455 promoter activity. (A) Schematic representation of the wild-type p455 reporter construct used in transfection experiments and position of the different deletion edges of the constructs used in (B). (B) cES cells were transfected with wild type or with one of the deleted p455 (p455 Del) luciferase reporters illustrated in (A). All luciferase activities were normalized by co-transfection with a CMV-renilla luciferase reporter. (C) The contribution of two putative Gata binding sites to the promoter activity of p455 was examined. Site directed deletions were performed in one of the following positions: Gata n°2: TATC -111/-114 or Gata n°3: TATC +47/+50. The deletion in site S1 described in Figure 1 and showing inhibition is used as reference. Luciferase activities obtained with these constructs in cES cells were compared to that obtained with wild type p455 as indicated in (B). Means are +/- s.d. of at least three independent experiments. Statistics are from t tests relative to the value obtained with p455. [file 1742-4690-9-21-S1.PNG]

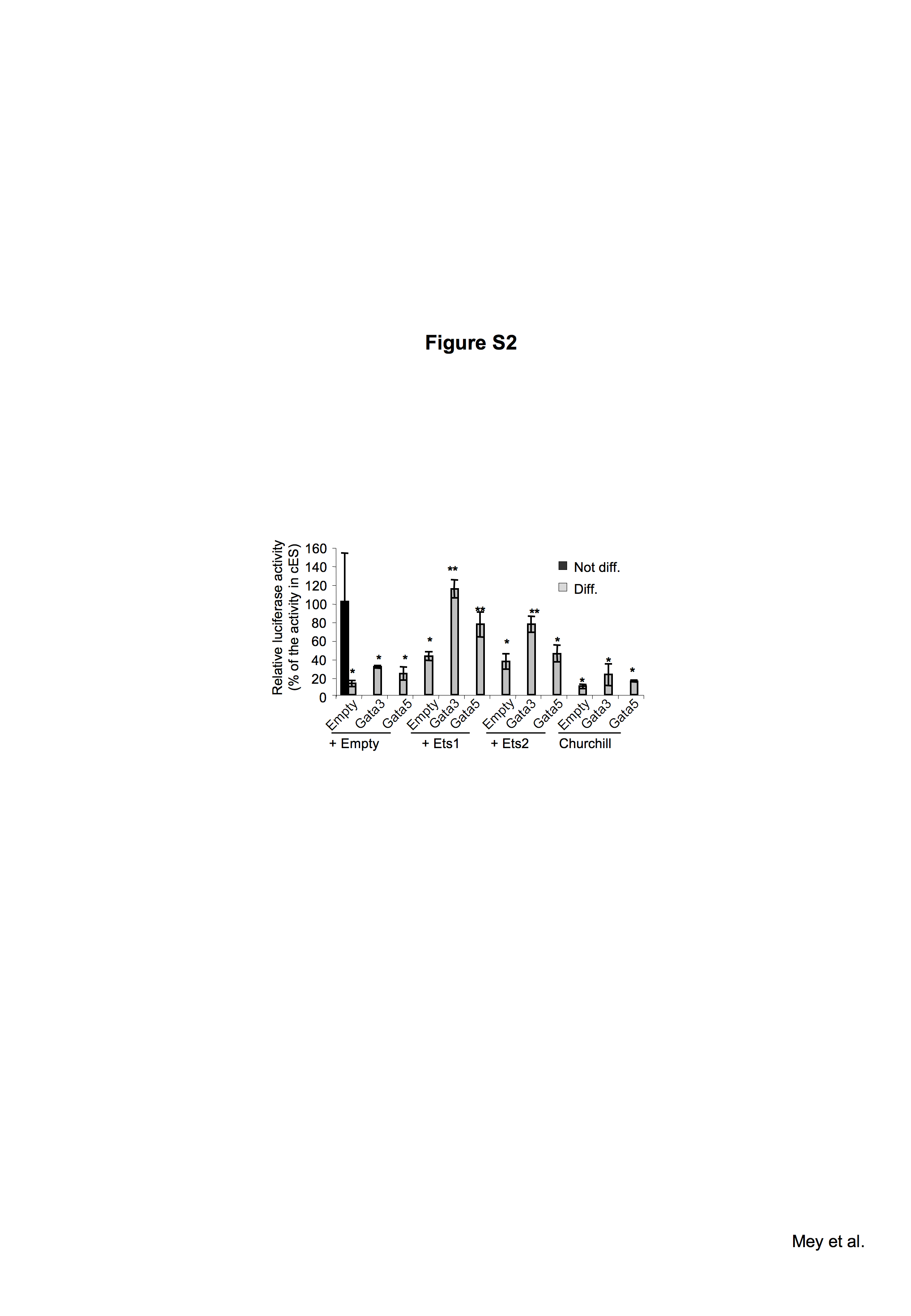

Supplement: Additional file 2 — Figure S2. Redundancy between Gata factors to restore the activity of the p455 promoter in differentiated cells. Experiments were performed as indicated in Figure 4 with equal quantities of pCi-neo vectors expressing the indicated transcription factors transfected in cES cells induced to differentiate 48 h with retonoic acid (Diff). Results are percentages of the value obtained with p455-Luc in cES cells transfected with empty vector. All the results are the means of three independent experiments +/- s.d. T test: *p < 0.05, **p > 0.05, relative to the values obtained in undifferentiated cells (Not diff.). [file 1742-4690-9-21-S2.PNG]

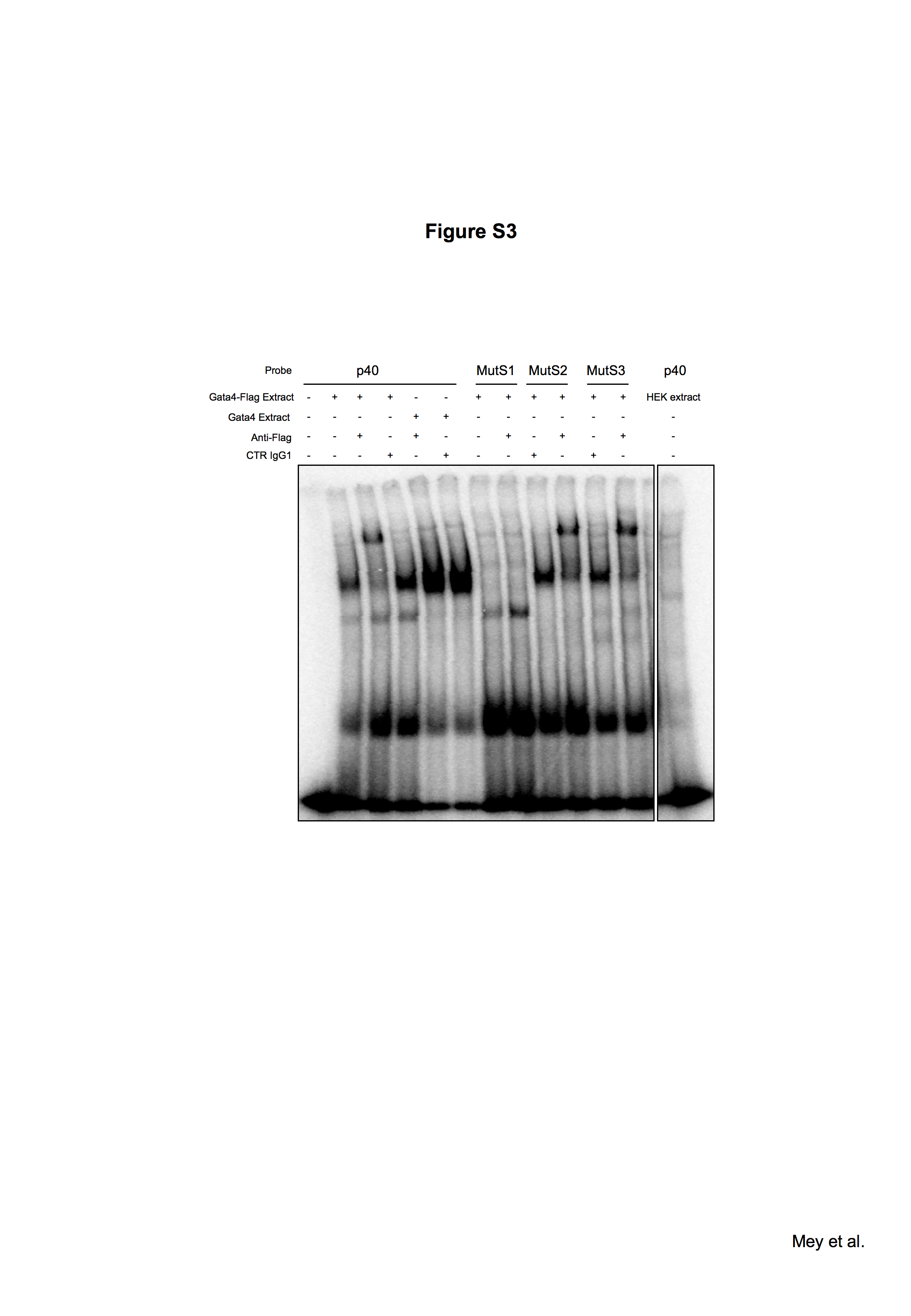

Supplement: Additional file 3 — Figure S3. Direct interaction of Gata4 with the site S1 of the promoter sequence. HEK293 cells were transfected with expression vectors encoding for the Gata4 protein in fusion with a flag tag or untagged as control. Nuclear extracts were used for EMSA assays with the labelled p40 probe or with p40 probes mutated in sites S1, S2 or S3. Supershifts were performed using an anti-Flag antibody or whole IgG as control; both used at 1 μg per lane. On the right is represented the result obtained with untransfected HEK293 cells. This lane is from the same gel but moved from the opposite side. Results are from one experiment representative of two. [file 1742-4690-9-21-S3.PNG]
